# Supplementary material for: Beliefs, compulsive behavior and reduced confidence in control
Source: PLoS Comput Biol. 2024 Jun 20;20(6):e1012207. doi: 10.1371/journal.pcbi.1012207 (PMC11218963; doi:10.1371/journal.pcbi.1012207)
Supplement: S1 Supplementary Material — Fig A. Results from Simulation 1B, otherwise Identical to Fig 3. Table A. Difference in world parameters and subjective beliefs (agent parameters), Δ params, for the compulsive and non-compulsive group in Simulation 1B (Mean (std)). Statistical comparison against 0 (no belief distortion) and between compulsive and non-compulsive group: bootstrapped t-test, d = Cohen’s d. * indicates significance. Table B. Regression between compulsion severity and the degree of belief distortion for doubt about washing effectiveness Δ pSUCCESS, overestimation of threat magnitude Δ costSICKNESS, overestimation of threat probability Δ pSOILING and underestimation of correct observations Δ pDETECT DIRTY and Δ pDETECT CLEAN based on Simulations 1A (full simulation of all parameters). * indicates significance. ρ is Spearman ρ. Table C. Regression between various aspects of compulsive episodes and the degree of belief distortion for washing effectiveness Δ pSUCCESS, overestimation of threat magnitude Δ costSICKNESS and overestimation of threat probability Δ pSOILING based on Simulation 2A, B, C (selective belief distortions). * indicates significance. ρ is Spearman ρ. Table D. Average belief (subjective probability of being in a dirty state) when performing each action in Compulsive and Non-compulsive agents based on Simulation 1A (full simulation of all parameters). Mean (Std). P-value from Bootstrapped U-test, d = Cohen’s d. Table E. Action transition probabilities for Compulsive and Non-compulsive agents based on Simulation 1A (full simulation of all parameters). Mean (std). P-value from Bootstrapped U-test, d = Cohen’s d. Table F. Absolute subjective beliefs (agent parameters) for the agents exhibiting pure checking (but no pure washing) compulsions, or pure washing (but no pure checking) in Simulation 1A and 1B (Mean (std)). Statistical comparison the pure checking and pure washing agents: Cohen’s d and bootstrapped t-test. * indicates significance. (DOCX) [file pcbi.1012207.s001.docx]

Supporting Information- Beliefs, compulsive behavior and reduced confidence in control

Lionel **Rigoux**^1,2^, Klaas E **Stephan**^1,2^, Frederike H **Petzschner**^3,4,5^

^1^ Max Planck Institute for Metabolism Research, Cologne, Germany

^2^ Translational Neuromodeling Unit, Institute for Biomedical Engineering, University of Zurich and Swiss Federal Institute of Technology Zurich, Zurich, Switzerland

^3^ Robert J. and Nancy D. Carney Institute for Brain Science, Brown University, Unites States

^4^ Department of Psychiatry and Human Behavior, Brown University, United States

^5^ Center for Digital Health, Brown University, United States

### Results for Simulation 1B: Relaxed checking constraint

This simulation of 10,000 agents differed from Simulation 1A in that it used a different criterium for compulsions and included agents whose policy did not require the 'check' action, allowing for the inclusion of agents who never performed checking behaviors (see Material and Methods - Simulations). The results of Simulation 1B are extremely similar to Simulation 1A (Fig 3 and Table 2 in the Main Text).

### Fig A


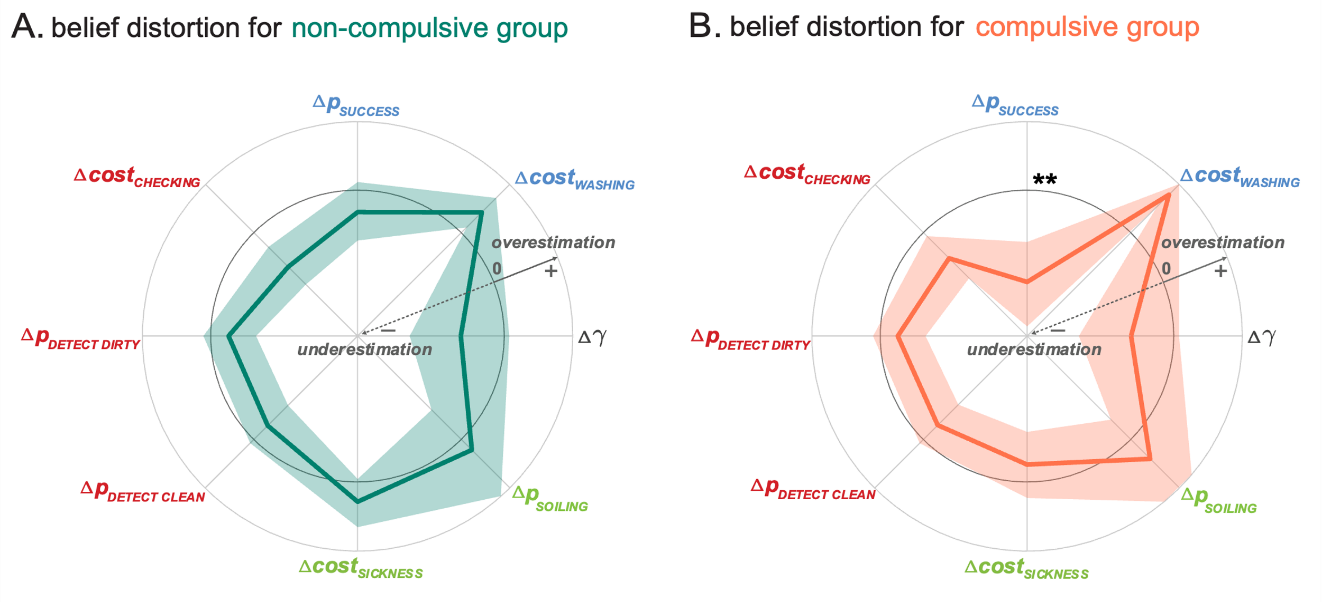


***Fig A.*** *Results from Simulation 1B, otherwise Identical to Fig 3 in the Main Text.*

*Table A*

|  | ***Non-compulsive*** | | ***Compulsive*** | | ***Group difference*** | |
| --- | --- | --- | --- | --- | --- | --- |
| ***Parameter*** | ***Estimate*** | ***t-test (p-value)*** | ***Estimate*** | ***t-test (p-value)*** | ***d*** | ***t-test (p-value)*** |
| $\Delta\gamma$ | -0.223 (0.343) | 0.047* | -0.219 (0.343) | 0.050* | -0.008 | 0.507 |
| $\Delta cost_{WASH}$ | 0.147 (0.163) | 0.007** | 0.276 (0.137) | <0.001*** | -0.890 | 0.061 |
| ${\boldsymbol{\Delta} \boldsymbol{p}}_{\boldsymbol{SUCCESS}}$ | **-0.114 (0.247)** | **0.138** | **-0.472 (0.285)** | **<0.001***** | **1.376** | **0.004**** |
| $\Delta cost_{CHECK}$ | -0.248 (0.186) | <0.001*** | -0.185 (0.211) | 0.005** | -0.291 | 0.366 |
| ${\Delta p}_{DETECT DIRTY}$ | -0.089 (0.184) | 0.132 | -0.089 (0.182) | 0.121 | -0.011 | 0.520 |
| $\Delta p_{DETECT CLEAN}$ | -0.099 (0.190) | 0.105 | -0.104 (0.191) | 0.093 | 0.023 | 0.497 |
| $\Delta cost_{SICKNESS}$ | 0.100 (0.192) | 0.107 | -0.091 (0.241) | 0.217 | 0.891 | 0.061 |
| ${\Delta p}_{SOILING}$ | 0.076 (0.427) | 0.413 | 0.141 (0.424) | 0.258 | -0.153 | 0.461 |
| ***Table A.*** *Difference in world parameters and subjective beliefs (agent parameters),*$\Delta params$*, for the compulsive and non-compulsive group in Simulation 1B (Mean (std)). Statistical comparison against 0 (no belief distortion) and between compulsive and non-compulsive group: bootstrapped t-test, d = Cohen’s d. * indicates significance.* | | | | | | |

### Relationship between false beliefs and compulsion severity

### We found that a distrust in the effectiveness of avoidance behaviors, here washing, led to increased severity of compulsions. This trend was not observed for any of the other belief distortions (see Table B).

### Table B

|  |  | $\boldsymbol{\Delta}\boldsymbol{p}_{\boldsymbol{SUCCESS}}$ | | $\boldsymbol{\Delta cos}\boldsymbol{t}_{\boldsymbol{SICKNESS}}$ | | ${\boldsymbol{\Delta} \boldsymbol{p}}_{\boldsymbol{SOILING}}$ | | ${\boldsymbol{\Delta} \boldsymbol{p}}_{\boldsymbol{DETECT} \boldsymbol{DIRTY}}$ | | ${\boldsymbol{\Delta} \boldsymbol{p}}_{\boldsymbol{DETECT} \boldsymbol{CLEAN}}$ | |
| --- | --- | --- | --- | --- | --- | --- | --- | --- | --- | --- | --- |
| ***Compulsion severity*** | | $\boldsymbol{\rho}$ | **p-value** | $\boldsymbol{\rho}$ | **p-value** | $\boldsymbol{\rho}$ | **p-value** | $\boldsymbol{\rho}$ | **p-value** | $\boldsymbol{\rho}$ | **p-value** |
| Percentage of agents with compulsion | | **-0.530** | **0.016*** | -0.399 | 0.081 | 0.087 | 0.473 | -0.018 | 0.502 | -0.095 | 0.467 |
| Number of compulsive episodes | | **-0.510** | **0.021*** | -0.382 | 0.097 | 0.054 | 0.474 | -0.016 | 0.521 | -0.050 | 0.494 |
| Duration of compulsive episodes | | **-0.555** | **0.011*** | -0.418 | 0.067 | 0.096 | 0.461 | -0.065 | 0.483 | -0.107 | 0.453 |
| Proportion of actions in a compulsive episode | | **-0.553** | **0.011*** | -0.423 | 0.063 | 0.087 | 0.462 | -0.076 | 0.470 | -0.088 | 0.464 |
| Belief update | | **0.725** | **<0.001***** | 0.356 | 0.123 | -0.033 | 0.492 | 0.077 | 0.460 | 0.146 | 0.422 |
| ***Table B.*** *Regression between compulsion severity and the degree of belief distortion for doubt about washing effectiveness* $\Delta p_{SUCCESS}$*, overestimation of threat magnitude*$\Delta cost_{SICKNESS}$*, overestimation of threat probability* ${\Delta p}_{SOILING}$ *and underestimation of correct observations* $\Delta p_{DETECT DIRTY}$ *and* $\Delta p_{DETECT CLEAN}$*based on Simulations 1A (full simulation of all parameters). * indicates significance.* $\rho$ *is Spearman* $\rho$*.* | | | | | | | | | | | |

### Table C

|  |  | $\boldsymbol{\Delta}\boldsymbol{p}_{\boldsymbol{SUCCESS}}$ | | $\boldsymbol{\Delta cos}\boldsymbol{t}_{\boldsymbol{SICKNESS}}$ | | ${\boldsymbol{\Delta} \boldsymbol{p}}_{\boldsymbol{SOILING}}$ | |
| --- | --- | --- | --- | --- | --- | --- | --- |
| **Compulsion severity** | | $\boldsymbol{\rho}$ | **p-value** | $\boldsymbol{\rho}$ | **p-value** | $\boldsymbol{\rho}$ | **p-value** |
| Percentage of agents with compulsion | | **-0.585** | **0.007**** | **-0.454** | **0.044*** | **0.449** | **0.047*** |
| Number of compulsive episodes | | **-0.603** | **0.005**** | **-0.457** | **0.046*** | **0.452** | **0.045*** |
| Duration of compulsive episodes | | **-0.606** | **0.005**** | **-0.459** | **0.042*** | **0.449** | **0.047*** |
| Proportion of actions in a compulsive episode | | **-0.606** | **0.005**** | **-0.458** | **0.042*** | **0.452** | **0.046*** |
| Belief update | | 0.421 | 0.066 | 0.004 | 0.507 | 0.226 | 0.305 |
| ***Table C.*** *Regression between various aspects of compulsive episodes and the degree of belief distortion for washing effectiveness* $\Delta p_{SUCCESS}$*, overestimation of threat magnitude*$\Delta cost_{SICKNESS}$ *and overestimation of threat probability*  ${\Delta p}_{SOILING}$ *based on Simulation 2A, B, C (selective belief distortions). * indicates significance.*$\rho$ *is Spearman* $\rho$*.* | | | | | | | |

### *Underestimation of avoidance success as a precursor to perfectionism/intolerance to uncertainty*

### Table D

| ***Parameter*** | ***Non - Compulsive*** | ***Compulsive*** | ***d*** | ***U-test (p-value)*** |
| --- | --- | --- | --- | --- |
| *Belief cook* | 0.252 (0.174) | 0.125 (0.123) | 0.885 | 0.011* |
| *Belief check* | 0.510 (0.201) | 0.471 (0.239) | 0.264 | 0.365 |
| *Belief wash* | 0.789 (0.175) | 0.739 (0.228) | 0.196 | 0.457 |
| ***Table D.*** *Average belief (subjective probability of being in a dirty state) when performing each action in Compulsive and Non-compulsive agents based on Simulation 1A (full simulation of all parameters). Mean (Std). P-value from Bootstrapped U-test, d = Cohen’s d.* | | | | |

## Compulsions are preventing exposure to true action outcomes

### Table E

| ***Parameter*** | ***Non - Compulsive*** | ***Compulsive*** | ***d*** | ***U-test (p-value)*** |
| --- | --- | --- | --- | --- |
| $\boldsymbol{p}_{\boldsymbol{COOK}\boldsymbol{\to}\boldsymbol{COOK}}$ | **0.445 (0.376)** | **0.187 (0.311)** | **0.678** | **0.023*** |
| $p_{WASH\to CHECK}$ | 0.306 (0.324) | 0.547 (0.432) | -0.563 | 0.120 |
| $p_{COOK\to WASH}$ | 0.249 (0.389) | 0.266 (0.432) | -0.039 | 0.443 |
| $p_{CHECK\to COOK}$ | 0.440 (0.284) | 0.449 (0.290) | -0.047 | 0.507 |
| $\boldsymbol{p}_{\boldsymbol{CHECK}\boldsymbol{\to}\boldsymbol{CHECK}}$ | **0.004 (0.060)** | **0.346 (0.337)** | **-1.042** | **<0.001***** |
| $\boldsymbol{p}_{\boldsymbol{CHECK}\boldsymbol{\to}\boldsymbol{WASH}}$ | **0.555 (0.285)** | **0.205 (0.173)** | **1.308** | **<0.001***** |
| $\boldsymbol{p}_{\boldsymbol{WASH}\boldsymbol{\to}\boldsymbol{COOK}}$ | **1.000 (0.001)** | **0.259 (0.421)** | **1.733** | **<0.001***** |
| $\boldsymbol{p}_{\boldsymbol{WASH}\boldsymbol{\to}\boldsymbol{CHECK}}$ | **0.000 (0.001)** | **0.537 (0.439)** | **-1.134** | **<0.001***** |
| $\boldsymbol{p}_{\boldsymbol{WASH}\boldsymbol{\to}\boldsymbol{WASH}}$ | **0.000 (0.001)** | **0.203 (0.318)** | **-0.594** | **0.002*** |
| ***Table E.*** *Action transition probabilities for Compulsive and Non-compulsive agents based on Simulation 1A (full simulation of all parameters). Mean (Std) P-value from Bootstrapped U-test, d = Cohen’s d.* | | | | |

## Relative costs cause differences in compulsion types: checking versus washing

### Table F

| ***Parameter*** | ***pure checking*** | ***pure washing*** | ***d*** | ***T-test (p-value)*** |
| --- | --- | --- | --- | --- |
| $\gamma$ | **0.826 (0.183)** | **0.569 (0.284)** | **0.933** | **0.019*** |
| $cost_{wash}$ | **0.286 (0.150)** | **0.115 (0.082)** | **1.445** | **0.001***** |
| $p_{SUCCESS}$ | 0.575 (0.277) | 0.347 (0.243) | 0.863 | 0.058 |
| $cost_{CHECK}$ | **0.167 (0.063)** | **0.368 (0.205)** | **1.027** | **0.002**** |
| $p_{DETECT DIRTY}$ | 0.797 (0.145) | 0.765 (0.146) | 0.209 | 0.427 |
| $p_{DETECT CLEAN}$ | 0.768 (0.141) | 0.760 (0.144) | 0.051 | 0.498 |
| $cost_{SICKNESS}$ | 0.547 (0.183) | 0.517 (0.201) | 0.130 | 0.465 |
| $p_{SOILING}$ | 0.379 (0.294) | 0.524 (0.288) | -0.472 | 0.240 |
| ***Table F.*** *Absolute subjective beliefs (agent parameters) for the agents exhibiting pure checking (but no pure washing) compulsions, or pure washing (but no pure checking) in Simulation 1A and 1B (Mean (std)). Statistical comparison the pure checking and pure washing agents: Cohen’s d and bootstrapped t-test. * indicates significance.* | | | | |
