# Supplementary material for: Beliefs, compulsive behavior and reduced confidence in control
Source: PLoS Comput Biol. 2024 Jun 20;20(6):e1012207. doi: 10.1371/journal.pcbi.1012207 (PMC11218963; doi:10.1371/journal.pcbi.1012207)
Supplement: Attachment — Submitted filename: Final_Response to Reviewers_PlosCB_OCDPOMDP.pdf [file pcbi.1012207.s002.pdf]

Response to Reviewers

First of all we would like to thank all reviewers for their exceptionally thoughtful and positive evaluation of the paper. It is extremely encouraging to read your assessments and we are deeply appreciative of your judgments and comments!

Below, we provide a detailed response to all the comments received. Green text indicates our response, blue text denotes the changes made in the paper. Page numbers are based on the Manuscript with Track Changes.

Reviewer #1:
This is a very interesting theoretical paper that shows that many aspects of OCD can be explained by a single parameter change in a cognitive computational model. The authors use a POMDP model of a cooking/handwashing/checking cleanliness scenario and many different simulations to investigations which parameters of the model might lead to compulsive behaviours (defined at both low and high thresholds), in scenarios in which the optimal behaviour did not include compulsions (due to the chosen parameters describing probabilities in the environment). They found that

- the only belief that distinguished compulsive from non-compulsive agents was a lower p(success) of handwashing than the actual p(success), and not other well-established hypotheses such as overestimation of threat magnitude/probability, or indeed the reliability of checking (or checking at all)
- the p(success) belief also affected the number, length and frequency of handwashing episodes
- although threat overestimation could trigger the onset of compulsive handwashing, only low p(success) was necessary and sufficient to sustain compulsions
- this core deficit then leads to an intolerance of uncertainty and perfectionism (e.g. in being extremely confident hands are clean before cooking)
- the relative costs of checking vs washing determine the proportions of these behaviours in the compulsions, however

The work is thorough and well-written and I support its publication - I have no major criticisms. Some relatively minor points are below:

Thank you so much for your thoughtful and positive feedback! We're thrilled to hear that you found our paper interesting and worthy of publication and didn't find major criticisms. Your insights and the time taken to review our work are greatly appreciated.

In Figure 5C, I thought that the compulsive/non-compulsive groups were defined according to whether washing/checking actions were repeated without cooking in between. If so, then aren't the differences in transition probabilities illustrated here there by definition? It seems odd to do statistical testing on the criteria for selection

to one group or another? It would make more sense to contrast groups with and without disparities between the believed and actual p(success).

Thank you for pointing this out. You are completely right that part of the results are expected and somewhat trivial, because the repetition of checks and washes is determined by the definition of compulsions. Yet there are other aspects of the transition probabilities that are not inherently tied to the definition of compulsions, including the asymmetries in transition probabilities. Specifically, the lower likelihood to transition from washing to cooking than from checking to cooking is not driven by the definition of compulsions, but by the distrust in p_success. In the manuscript, we have now clarified what is expected and what is novel:

p24: "As compulsive behaviors are characterized by the repetition of certain action patterns, we next examined the probabilities of transitions between actions, such as from washing to cooking (Figure 5C). As expected from our definition of compulsions, agents in the compulsive group were significantly more likely to repeat preventive actions like checking or washing (compulsive group: = 0.35 (0.34), $p < 0.001$, = 0.20 (0.318), $p = 0.002$; non-compulsive group: = 0.004 (0.06), = 0 (0.001), see bootstrapped U-tests in Supporting Information - Table S5).

In addition, while non-compulsive agents typically resumed cooking immediately after washing their hands (NC: = 1 (0)), compulsive agents often engaged in additional checking or washing actions before returning to cooking (C: = 0.26 (0.42), $p < 0.001$). Notably, this behavioral reorganization was not observed for checking: both groups were equally likely to resume cooking after checking. This asymmetry is likely driven by the distrust in the washing actions."

p24 - it doesn't make sense to me to say "It implies that compulsive agents are less frequently exposed to the consequences of their washing actions". Compulsive agents are much more likely to check their hands are clean after washing than non-compulsive ones? This is surely exposure to the consequences of washing?

You are completely right, this was an unfortunate formulation. We removed the sentence and rephrased this part. What we meant is that the consequences of cooking after washing are much less frequently observed than the consequences of cooking after checking. So outcomes of actions one step later might pose an important challenge for credit assignment because successful cooking may indeed be more likely assigned to having checked (as a preventive action) rather than having washed.

p24: "This implies that while controls experienced the reward of having cooked properly always right after washing their hands, compulsive agents were exposed to this reward only after having first checked —possibly multiple times—, thus stretching the link between washing and its positive outcome, which complicates credit assignment."

p38 - The authors draw a distinction between their proposal and the recent paper by Fradkin et al, saying that the latter merely propose greater uncertainty over all

state transitions, whereas they more specifically propose that compulsions result from state-action transitions relating to harm prevention. I'm not sure these proposals are really so far from each other, however, as in the Fradkin paper those authors discuss action-dependent transitions in particular in the context of compulsion formation, and in this paper (on the next page) the authors cite experimental work showing that OCD patients are impaired learning about transitions of seasons (which are presumably not action-dependent?). So really it is the balance of general vs specific deficits that tilts slightly different ways in these papers. To me the biggest difference between the papers is the robustness of the analysis here: they have done thousands of simulations demonstrating both sufficiency and necessity of the action-dependent belief in causing compulsions (and excluding other potentially responsible parameters).

Thank you for pointing this out. You are fully correct that the concepts are related. In Fradkin et al. one issue is that uncertainty is non-specific and applied to both action-dependent as well as independent state transitions. The interesting insight that our simulations show is that action-dependent distrust is very specific to one specific type of actions, e.g. patients do not have distorted assessment of p_soiling or checking and that this unique distorting can be the root cause of state uncertainty instead of being two phenomena that occur in parallel. We are now making this part more explicit:

p.39: "Our model offers a nuanced expansion of the concept of state uncertainty, particularly in its relevance to OCD. We find that the form of uncertainty pivotal to OCD is specifically linked to state-action transitions concerning harm prevention. This focus is distinct from a broader, generalized uncertainty about state transitions or other kinds of action-state interactions, such as those related to incidental contamination (e.g. likelihood of contaminating hands while cooking).

Furthermore, our simulations show that compulsions in OCD are not propelled by a uniformly elevated level of uncertainty around these action transitions. Instead, they stem from a precise, asymmetrical underestimation of the success in harm avoidance activities. This further suggests a context-specificity of compulsions for OCD that are specific to preventive actions for harm avoidance.

Crucially, we show that this unique underestimation of successful harm prevention can serve as the root cause for other forms of uncertainty and doubts, including causing uncertainty in state transitions as they have been proposed in Fradkin et al (see Figure 5B and Figure 7)."

p41 - The authors comment on the elevated sense of responsibility in OCD but say their model stops short of explaining this phenomenon. Reading this I wondered whether an asymmetry in the belief about the efficacy of preventative actions (i.e. doubting actions that prevent harm more than those that cause harm) does in fact account for this? After all, we all have urges to do things that we then terminate at some stage of planning/execution. Most of us can be confident that a) we could perform such an action, and b) we could stop ourselves from performing the action.

If these can be regarded as separate actions, then for an OCD patient, presumably just imaging themselves doing something bad would inevitably make that action more likely, as they believe they are less able to prevent it than to execute it? This must lead to a great sense of responsibility over what one even contemplates. The authors are under no obligation to include these speculations however!

This is a very interesting thought that sparked a spirited discussion among us. We think you might be right. We mention this option in the discussion now as a speculation:

p. 43: "One speculative thought could be that believing that one is unable to prevent bad outcomes makes individuals more likely to believe that they will cause harm, which results in a sense of responsibility. How responsibility is linked to beliefs about harm prevention remains an open area for future investigation, offering potential insights into the complex cognitive mechanisms underpinning OCD."

Reviewer #2:
In this paper, Rigoux et al, propose a quantitative modeling approach of Obsessional-Compulsive Disorder (OCD), by simulating the behavior of human agents described by a limited set of parameters characterizing their beliefs formation process in a simplified paradigmatic symptom-triggering situation (handwashing) which the agents are repeatedly exposed to, under a normative learning decision scheme (Partially Observable Markov Decision Process, POMDP) that dictates their evolution. By systematically varying agents' parameters' values, Rigoux et al observe that the critical trait of agents exhibiting compulsive behaviors in this scenario (and the main factor associated with the intensity of such compulsive behaviors) was the underestimation of the effectiveness of one's threat-eliminating action (here cleaning) in contrast to other dysfunctional beliefs pertaining to, for example, overestimating the danger associated with dirt, being hypersensitive to dirt, etc. all of which are hypothesized core dysfunctional beliefs according to the most prevalent psychological theories of OCD. In addition, having such dysfunctional beliefs about the outcome of one's action nurtured a less efficient belief updating in the simulated agents, which authors associate with the exacerbated uncertainty and pathological doubt self-reported by OCD patients.

This computational psychiatry simulation study represent a very welcome endeavor to confront multiple hypotheses regarding the cognitive roots of OCD focussing on the dysfunctional beliefs that may operate in the decision-making process of patients. Empirical studies fail to arbitrate between these theories which make a number of common predictions, though not quantitative ones. That is where a modeling work like this one represents an insightful progress by directly comparing the consequences of each of these hypothetical core dysfunctional beliefs as they are implemented in a population of evolving simulated agents under the exact same circumstances. My opinion is that more work of that quality should be published as it would help moving the field of cognitive psychopathology forward.

Thank you very much for your positive feedback! It is extremely encouraging to see that you value our approach and we wholeheartedly agree that the inspiration behind this work was to use a modeling framework to discern different hypotheses that can typically not easily be tested experimentally.

Obviously, the numerical tracking of these agents requires the authors to restrain themselves to a simplified scenario and to assume each agent to obey optimal decision making under uncertainty. And this may feel like oversimplification to the clinically-minded reader. Yet the authors make a great effort to translate, ex ante, how clinical hypotheses might be subsumed in specific design choice of their model and, ex post, the meaning of the outcome of the simulations. Yet I feel that on critical aspect that may escape the reader unfamiliar with such modeling work is that the modeled agents are plunged in an iterated (or sequential) learning framework under which they adjust their beliefs about the world through repeated instances of actions and outcomes.

You are absolutely right that we could emphasize more that our agents are in fact updating their belief as they choose actions. We are now emphasizing this more at the beginning, when we are introducing our model.

page 8: "In this specific scenario, agents are preoccupied with the possibility of transmitting germs from their hands to the food, which could potentially result in illness for their guests (Figure 2). This concern requires them to constantly reassess their situation based on their actions and observations: deciding whether it's safe to cook given their current perception of hand cleanliness, or whether they should engage in behaviors like washing their hands or checking them for cleanliness. Agents are thus constantly updating their beliefs about whether the hands are dirty or clean."

The authors also suggest a number of empirical predictions that could be derived from their in-silico results. This is also very much commendable as this effort is only too rarely made by authors in computational works, including computational psychiatry research.

Thank you very much!

Having little to critique on the heart of the simulation work, I feel that the discussion should expand on an important though not critical issue: As acknowledged by the author themself, the agent model they deploy does not allow the parameter to change and adjust (e.g. as could be achieved in a Bayseian framework as suggested by the authors themselves). As a consequence, their model can not inform on the onset of OCD while a number of clinical presentations seem to appear in the young adults with little if any earlier signs or symptoms. In my opinion the other should discuss how their modeling work speaks to a developmental / trait disorder and how to interpret life-long learning with respect to their sequential framework.

Thank you for raising this point. Indeed, due to the nature of the model, we cannot say much about the developmental aspects of the disorder. However, we have now added a section in the limitation section that speaks to this point and speculates on a potential avenue for resolution.

p44: “Finally, our model does not describe how those individual beliefs about contingencies in the world (agent parameters) are acquired. This means it cannot discern between different developmental aspects of OCD, e.g. explain differences in onset and comorbidity between pediatric and adult OCD (83). While a Bayesian reformulation of our model could potentially help capture such dynamics, we can at this point only speculate that during childhood and early adolescence a model of the contingencies of the environment is forming that becomes more rigid with age.”

Finally, the authors should be commended for making all code, data and intermediate computing state, down to figure rendering freely and easily available on their github. However, I must say the high-performance computer resources required to re-run the analyses limit my ability to actually track the full extent of the data.

We are glad to see our efforts of transparency and reproducibility appreciated. For the sake of the paper, we indeed opted for large scale computations, a full grid of 2,000 actions x 100 agents x 100 worlds, to be as accurate as possible. There is, however, a way to recreate the gist of the results (with less accuracy) by running the simulations on a much smaller grid, e.g. 200 actions x 10 agents x 10 worlds, which is more tractable. We have adapted the code to run such a “minimal” example by default while providing guidance to upscale the simulation as described in the manuscript. We now also offer a small toy example for users to play with an illustrative example without having to run the full-scale simulation and analysis pipeline.

Reviewer #3:
This is a very interesting paper, I very much enjoyed reviewing this. The paper uses a computational modelling approach, based on POMDPs to identify through simulations what could cause compulsive and obsessive symptoms in OCD. They find both expected (a faulty believe in how likely handwashing is to clean hands leads to compulsive behaviour) and novel results (beyond these believe abnormalities, no further changes needs to be made to the effectiveness of checking to produce compulsive behaviour). Interestingly, they can also use their model to explain previously reported findings (learning deficits in OCD) that were not explicitly included in the model. The code accompanying the paper is freely available and annotated.

Thank you for your positive assessment and helpful input!

I only two one ‘major’ comment (but they are pretty small points of criticism actually):

1. Can your model also be fit to agents to test parameter recovery? If not, how would you use your paradigm in a study of actual participants? (You say in the online code annotations that some of it took weeks to run on a cluster) E.g. if you had data from real participants in a task, would you be able to estimate whether their behaviour is due to uncertainty about washing success and checking success? Please could you say something about this in the discussion.

Thank you for pointing this out. You are indeed right that the code had been running for several weeks, but we aimed for extensivity by covering the full space of parameters in a large set of agents and worlds (2000 actions x 100 agents x 100 worlds). For an actual experiment, one could decide to focus on a specific world (set of world parameters) carefully selected to elicit an optimal strategy particularly sensitive to the parameter of interest, and collect a much smaller number of trials per participant, e.g. 200. While the model inversion would still require exploring the policy space, this would significantly reduce computing time and make the model inversion much more feasible.

In terms of parameter recovery, we are confident that we could recover the parameter of interest p_success in such a scenario because of the uniquely associated behavior patterns identified in the manuscript. However, it is unclear if all other parameters could be fully identified in such a short task. For example, empirically dissociating the relative influence of the two observation probabilities p_detect_clean and p_detect_dirt might not be achievable in a realistic time-frame. To check this, one would need to run simulations of the task under the specific choice of world parameters and assess for each parameter the quality of the recovery.

Yet another alternative could be to test two versions of the task, one where avoidance actions can be selected by the participant (active prevention), and one where they are e.g. selected by a computer. Comparison of the respective policies would allow us to assess how strongly belief updates are tied to assessments of one's own action.

We have now added a section in the discussion that addresses that topic:

p.41: "To directly assess beliefs regarding successful harm prevention, one could develop a dedicated task based on the simple handwashing scenario outlined in this study or a similar task design (80). To ensure empirical feasibility and computational tractability, it would likely be necessary to select a specific instance of world contingencies (world parameters) that optimize parameter recovery for the limited number of trials that can be acquired. While this paradigm should effectively uncover beliefs associated with successful harm prevention (p_success), thorough simulations would be required to evaluate parameter recovery across the various other parameters.
In addition to implementing the proposed paradigm, one could also introduce a variant where avoidance actions are either actively chosen by participants or selected

by a different agent. Comparing policies between these two paradigms would provide valuable insights, as discussed earlier."

2. It is intriguing that (figure 3), compulsive agents differ in their estimation of being able to successfully clean, rather than e.g. their ability to check. Your discussion of this and relating it to the phenomenology of 'not-just-right' is very interesting. Do you find that when analysing the behaviour of these agents that they do have also an increase of check actions, or do they just differ in their washing actions? Would it be possible to somehow illustrate the raw behaviour, e.g. something similar to figure 2B but across the different agents? This is somewhat addressed in figure 4A, but if I understand correctly, this is not separate for episodes defined based on checking or on washing? Somewhat conceptually related, in recent data in a large sample by Trier et al. (2023, preprint), there was no difference for checking actions with a transdiagnostic compulsivity dimension, but there were changes to active avoidance behaviour. (However, that data were not modelled with POMDP, so it is not directly comparable unfortunately).

Thank you for this point. Indeed, we observed different patterns of repetitive checking and washing. We have a section on it in the Results and in Figure 6, which we now highlight in the discussion as well. More precisely, while some agents just predominantly checked, others predominantly washed, and yet others show a very mixed pattern. Taking a closer look at what determines the compulsion pattern reveals that the relative costs of the actions that determine the most likely action sequences. For example, if checking is more costly, the agent more likely engages in repeated washing (as opposed to repeated checking) when they think their hands are dirty. Interestingly, while the probability of successful washing determines the compulsion severity, it did not affect the exact pattern which was rather determined by the cost of actions. This is illustrated in Figure 6. Because the exact pattern depends on the associated costs only, we decided that it's clearest to show every participant as a dot in this 3-D cost space to illustrate how the individual patterns come about.

In Figure 6 legend it says: "*Agents exhibiting pure washing compulsions (blue dots) all had a low cost of washing (left side of the simplex), while agents exhibiting pure checking compulsions (red dots) all had a relatively low cost of checking (right side). When the cost of washing and checking are balanced, agents were characterized by mixed compulsions (purple dots).*"

And we discuss it in the Results section: "Relative costs cause differences in compulsion types: checking versus washing"

p24-26: "Compulsions tend to cluster in different dimensions, such as checking, washing, ordering. In our framework we could analyze the individual trade-off between two of these behaviors, checking and washing. A common observation across all our simulations was that an underestimation of the effectiveness of preventive actions invariably led to compulsive episodes. While the degree of underestimation scaled with compulsion severity (in terms of probability, length, and frequency of episodes),

the relative proportions of the different compulsion types (only checking, only washing, or a mix of both) remained consistent.

However, a comparison between different simulations that employed different criteria for compulsions (simulation 1A: requiring agents to perform all actions and simulation 1B: where checking was not mandatory; see Simulating compulsive and non-compulsive agents) revealed notable differences in compulsive behavior patterns. In simulation 1A a significant proportion of agents showed pure checking or mixed compulsions, with only 37.2% demonstrating pure washing compulsions. In contrast in simulation 1B the vast majority of agents (97.7%) exhibited pure washing compulsions.

To understand what explains the emergence of checking vs. washing compulsions, we analyzed the subjective costs attributed to these actions by agents in both simulations. We found a distinct pattern: agents exhibiting pure checking compulsions perceived a relatively lower cost of checking, while those with pure washing compulsions perceived washing as less costly. Agents who assigned similar costs to both actions tended to exhibit mixed compulsions (Figure 6 and Supporting Information - Table S6). This finding indicates that the specific costs assigned to actions like checking, washing, or cleaning play a pivotal role in determining the nature of compulsive behavior, independent of the agent's confidence in their actions or the severity of symptoms."

We have also added a sentence in the Discussion, where we originally discussed the not-just-right feelings and are citing:

"Our simulations showed that when agents were uncertain as to whether their handwashing was effective, it took multiple actions (washing and checking) to change their belief from thinking their hands were dirty to thinking they were clean (see Equation 2 and Figure 5B), whereby the exact pattern of washes and checks was determined by the individual costs of each action (Figure 6)."

Thank you for pointing out the Trier paper, we are now citing it in our discussion section when we point to concrete implementations of this paradigm.

p.41: "To directly assess beliefs regarding successful harm prevention, one could develop a dedicated task based on the simple handwashing scenario outlined in this study or a similar task design (80)."

Indeed, this experiment from the Trier Paper could in principle be modeled the POMDP presented here, with minor adaptations (e.g. time discretization). In fact, in the Trier paper the "hide" action is quite similar to the "wash" action in our case: it prevents the negative outcome of the "forage" action which depends on the presence or not of a predator (the hidden state), whereby a longer repetition of hide actions after the predator left could be linked to increased state uncertainty, like the compulsive washing we observe in our case. Indeed, in the Trier paper they find that individuals with high compulsive traits wait longer than necessary before exiting the hiding place. The absence of an effect of checking could be explained by the cost

function, although this would require a more detailed simulation of the exact task structure.

Here are my minor comments, almost all of these refer to specific places in writing (mainly methods) where some clarity could be added:

3. The authors put their code online. The code is very tidy and well annotated inside the code. However, if there was something like a tutorial walk through, I wonder whether this would make this approach easier for other people to implement in their studies? E.g. something that would link the specific aspects described in the methods to the code.

Thank you for taking the time to explore and provide feedback on our code. We agree that it required an easier entry point. Following this suggestion, we added a section on the landing page explaining how to run a minimal example (create a world, simulate an agent with distorted belied, diagnose behavior). We also adjusted the main script to run a reduced set of simulations that should run without the need for a computing cluster.

4. In the methods you say on page 47 "In principle, this can be numerically computed, using for example, dynamic programming." Just to clarify, do you mean that you did this using dynamic programming? Or are you saying you could have done that, but you did something else?

We apologize for the ambiguous phrasing. We did use dynamic programming, as clearly stated in the revised manuscript now:

p. 48: "In our case this was computed numerically using dynamical programming methods (85,86) (see below)."

5. Reading table 4, I did not quite realize what is a parameter. And I also did not quite understand what is meant by parameter – e.g. in a decision making task it would be 'sensitivity to reward'. Could I clarify that here then parameter would mean e.g. for cost_sickness 'how sensitive the agent is to the cost of sickness' – I think I got confused because I assumed that this was determined by the environment. Maybe please clarify what is a characteristic of the environment – and thus set by the experimenter – and what is instead a property of how the agent perceives the environment.

Thank you for pointing this out. Sensitivity to reward can indeed introduce a bias between the true utility of an action and its subjective appreciation. Typically, in experimental studies, the experimenter controls the objective value, while the weight (i.e., reward sensitivity) that maps the objective value to the subjective value becomes the parameter of interest for measurement. In our model, we denote the objective utility as a "world parameter" and the subjective utility (i.e., the objective utility weighted by the agent's sensitivity) as the "agent parameter." In our simulations, both parameters are varied independently, hence the term "parameter." While the ratio between subjective and objective utility could potentially recover the

agent's sensitivity, we encountered difficulties in generalizing this approach to other aspects of the task structure due to a lack of terminology that applies universally (e.g. it seems inadequate to call "sensitivity to cleanliness" an increase in the probability of correctly detecting that the hands are clean). For simplicity, we solely refer to objective and subjective values as parameters in our analysis.

We have revised the manuscript to provide clearer definitions of the various parameters used.

p. 49: "*Each action is associated with pay-offs, as captured by a reward function R. Importantly, we consider here that this reward function reflects the true utility of actions which might differ from the one perceived by the agent due to its idiosyncratic sensitivity to costs and benefits (but see below Modeling action sequences for individual agents).*"

p. 54: "*For example, an agent who is overly sensitive to threats, and thus overestimates the expected cost of cooking with dirty hands, will be modeled with an agent parameter $cost_{sickness}'$ that is larger than the objective cost (world parameter) $cost_{sickness}$.*"

6. In table 5, I don't understand why the probability for observing 'clean' is 50% when cooking or washing. I would have assumed that – in the absence of checking – this would instead be a free parameter, something like capturing your belief that because you have cooked, it must be dirty now (maybe combined with a prior belief about whether your hands were already dirty). Or no update to the believes at all in the absence of a check action. I don't quite understand what the 50% is meant to capture instead.

The reviewer's intuition is indeed accurate and aligns with the implementation of the model in Eq.2: the prior belief about the hands' dirtiness (b_t) is combined with the estimated probability of getting dirty while cleaning (the agent's parameter p_soiling'). However, as per the POMDP formalism, each action must be associated with an observation (o_t), which also influences the belief update. While this association is reasonable for checking actions, as pointed out by the reviewer, in scenarios such as cooking or washing, observations are not expected to contribute to belief updates.

To address this within the POMDP framework, a common approach is to set the observation to chance level (50%). This effectively renders observations non-informative, allowing them to be disregarded during belief updates. Formally, by setting $O(o_t|a_t, s_{t+1}) = 0.5$ in Eq.2, the observation can be completely factored out of the belief update rule.

We now explicitly describe this approach in the manuscript.

p.51: "*In our model, we included two observations, W = {dirty, clean} which are only informative for the check action (Table 5); for the wash and cook actions,*

observations are evoked at chance (0.5) and are therefore uninformative, i.e. while defined, they effectively do not affect the belief update (see below Eq. 2).”

7. On page 55, when talking about optimal policy, please could you clarify whether the policy was optimal given full knowledge of the world? Or given subject parameters that mapped correctly onto the world, or what exactly was optimal – e.g. maximum rewards achieved empirically?

We agree that our phrasing was confusing. We have rewritten the paragraph to clarify that optimality is to be understood relative to the agent’s subjective representation of the world:

p. 56: “*In the previous section, we mentioned that the sequence of decisions depended both on the subjectively optimal policy (the solution of the POMDP according to the internal representation of the world by the agent) and the true contingencies of the world (the actual state transitions and observations).”*
